# 小细胞肺癌分子分型研究进展

**DOI:** 10.3779/j.issn.1009-3419.2021.101.36

**Published:** 2021-10-20

**Authors:** 梦圆 徐, 俊文 张, 延军 苏, 西川 李

**Affiliations:** 1 300387 天津，天津师范大学生命科学学院，天津市动植物抗性重点实验室 Tianjin Key Laboratory of Animal and Plant Resistance, College of Life Sciences, Tianjin Normal University, Tianjin 300387, China; 2 300060 天津，天津医科大学肿瘤医院肺部肿瘤科，天津市肿瘤防治重点实验室，天津市肺癌诊治中心 Tianjin Lung Cancer Center, Key Laboratory of Cancer Prevention and Therapy, Department of Lung Cancer, Tianjin Medical University Cancer Institute and Hospital, Tianjin 300060, China

**Keywords:** 肺肿瘤, 分子分型, 疾病治疗, 诊断, Lung neoplasms, Molecular typing, Disease treatment, Diagnosis

## Abstract

小细胞肺癌（small cell lung cancer, SCLC）是一种极具侵袭性和致命性的恶性肿瘤，具有病因复杂、分化程度低、恶性程度高、生长速度快、侵袭性强、转移早和获得性耐药等特点，导致患者的预后普遍较差。近年来，随着人们对SCLC发生发展分子机制研究的逐渐深入和多组学数据的深入挖掘，提出可以按照细胞内关键转录因子的差异表达进行分子分型，包括SCLC-A、SCLC-N、SCLC-P和SCLC-I等亚型。对SCLC进行分子分型研究并应用于临床，将有助于提高医生对SCLC患者的详细诊断和治疗方案的进一步优化，从而延长患者生存时间，提高患者生活质量。

## Abstract (en)

Small cell lung cancer (SCLC) is a highly aggressive and fatal malignant tumor. It has the characteristics of complex etiology, low differentiation, high malignancy, fast growth, strong invasiveness, early metastasis and acquired drug resistance, resulting in poor prognosis. In recent years, with the gradual deepening understanding on the molecular mechanism of SCLC and multi-omics data, it is proposed that molecular typing can be carried out according to the differential expression of key transcription factors, including SCLC-A, SCLC-N, SCLC-P and SCLC-I subtypes. Molecular typing of SCLC and its clinical application will help doctors to further optimize the detailed diagnosis and treatment plan of SCLC patients, so as to prolong the survival time and improve the quality of life of patients.

肺癌是当前世界范围内最常见的恶性肿瘤之一。最新全球癌症数据^[1]^显示，2018年肺癌的发病率与死亡率分别为11.6%和18.4%。在中国，肺癌的发病率占所有癌症病例的21.9%，位居所有恶性肿瘤发病率第一位^[2]^。其中小细胞肺癌（small cell lung cancer, SCLC）约占所有肺癌病理亚型的15%^[3]^。

## 1 SCLC的基本特征

SCLC是一种极具侵袭性和致命性的恶性肿瘤，与非小细胞肺癌（non-small cell lung cancer, NSCLC）相比，具有倍增速度快、恶性程度高、较早发生广泛转移、易伴发内分泌紊乱综合征、初次化疗敏感但易复发且获得性耐药、瘤内血管密度高、失巢凋亡抵抗、基因组不稳定和多种信号通路失调等特征，总体预后极差^[4, 5]^。

根据临床分期，SCLC可分为局限期（limited-stage, LS）和广泛期（extensive-stage, ES），其中LS期患者约占所有SCLC患者的1/3，患者中位生存期（median survival time, MST）一般为15个月-20个月，而大部分患者在确诊时已处于ES期，生存期一般为9个月-12个月，平均2年生存率低于10%^[6, 7]^。当前对SCLC患者的治疗一般不行外科手术，主要是以放疗与化疗相结合为主^[6]^，多数ES-SCLC患者在放化疗结束的1个月-3个月后出现复发，且复发后出现对原化疗药获得性耐药，通过剂量强化难以获得改善^[8]^。

目前对SCLC的诊断主要依靠血液肿瘤标志物检测、计算机断层扫描（computed tomography, CT）等影像学检测和穿刺标本的病理学检测来判断。SCLC细胞主要的形态学特征包括燕麦状小细胞、胞质稀少、核质明显、边界不清、胞内具有颗粒状染色质、缺乏明显的核仁等。肿瘤呈高分级、高增殖、高凋亡、高坏死，组织Ki-67染色阳性率为50%-100%^[9]^。

## 2 SCLC的分子分型

在几乎所有SCLC病例中，抑癌基因*TP53*和*RB1*的失活普遍存在，表明*TP53*和*RB1*的双失活是SCLC发生的必要条件之一^[10]^。由于*TP53*和*RB1*的普遍缺失和细胞的神经内分泌上皮分化，SCLC最早被认为是分子同源的。随着研究的深入，人们逐渐发现不同的SCLC肿瘤间存在异质性，甚至同一个SCLC患者的不同肿瘤细胞间也可能存在肿瘤内异质性，因此将SCLC分为经典型和突变型。随后，根据肿瘤细胞是否具有神经内分泌状态特征，将其分为神经内分泌型（neuroendocrine, NE）和非神经内分泌型（non-neuroendocrine, non-NE）。近年来，人们根据人原发性肿瘤组织、癌细胞系、PDX小鼠移植瘤和基因工程小鼠（genetically engineered mouse model, GEMM）的大量多组学测序数据和SCLC发生发展分子机制的深入研究，提出按照SCLC细胞内转录因子的表达差异进行分子分型。

Rudin等^[9]^分析了已发表的81例手术切除的、大多为LS-SCLC肿瘤的RNA-seq数据，通过归类分析发现SCLC的分子亚型可以由转录因子ASCL1、NEUROD1和POU2F3的差异性表达及其伴随的炎症基因表达来定义^[11, 12]^。通过鉴定上述3种转录因子和炎症基因的表达差异，可以将其区分为SCLC-A（高表达ASCL1）、SCLC-N（高表达NEUROD1）、SCLC-P（高表达POU2F3）和SCLC-I（3种转录因子均低表达，但炎症基因高表达）四种分子亚型。

### 2.1 SCLC-A

生理状态下，转录因子ASCL1在神经干细胞等细胞中高表达，可诱导神经元和中性粒细胞的分化。病理状态下，*ASCL1*是一个特异性的癌基因，可以在SCLC细胞内表达，是高级别SCLC的潜在治疗靶点^[9]^。因此，将高表达ASCL1的SCLC亚型定义为SCLC-A。

ASCL1的表达与DLL3的表达密切相关，DLL3是编码抑制Notch信号通路、抑制神经元和NE细胞分化的抑制剂，并与REST的表达缺失密切相关。在SCLC中Notch通路的内源性激活导致10%-50%的肿瘤细胞发生NE到non-NE的分化。该分化的起始部分由抑制NE基因表达的转录抑制因子REST/NRSF介导^[13]^。

### 2.2 SCLC-N

大部分的SCLC会表达ASCL1和NeuroD1两种转录因子中的一种或两种，其中约15%的SCLC细胞系和肿瘤会不表达ASCL1，只表达NeuroD1。因此将不表达ASCL1、只表达NeuroD1的SCLC亚型定义为SCLC-N。

NeuroD1的表达通常与ASCL1相关，但是这两者对NE细胞有着不同的的靶向功能^[9]^。ASCL1靶向癌基因*RET*、*SOX2*和*NFIB*以及*DLL3*和*DLL1*等Notch通路中的多个基因；而NeuroD1则靶向MYC。在SCLC中会出现从ASCL1^+^转变到NeuroD1^+^的现象^[14]^。这种ASCL1表达缺失在复发肿瘤中更为常见。在GEMM小鼠实验^[15]^中发现，在MYC过表情况下，SCLC的GEMM肿瘤会表现出从ASCL1^+^/NeuroD1^-^的经典形式到ASCL1^-^/NeuroD1^+^的变异形式的快速转换现象，并伴有NE细胞标记物丢失的现象。NEUROD1在非内分泌肺癌细胞系中过表达会导致细胞增殖并激活NE转化程序，且影响SCLC-N细胞的迁移能力^[16]^。此外，在SCLC细胞中的转录因子扩增方面，SCLC-A细胞系主要是MYC1扩增，具有典型的SCLC形态；SCLC-N细胞系则主要是*MYC*扩增，具有变异特征^[16]^。虽然在内分泌SCLC中ASCL1与NeuroD1是必须的转录因子，但在高级别肺NE瘤小鼠模型中，ASCL1相比于NeuroD1更为重要。

### 2.3 SCLC-P

Rudin等^[9]^的研究鉴定出过表达POU2F3的SCLC亚型，发现其是一种非神经内分泌型、非去甲肾上腺素（Non-NE、Tuft-cell）变体，将其定义为SCLC-P亚型。

生理状态下，POU2F3通常在簇绒细胞中选择性表达，而簇绒细胞是肺上皮中一种罕见的化学感应细胞类型^[17]^。病理状态下，POU2F3一般在缺乏高水平ASCL1或NeuroD1表达的SCLC细胞系亚型中表达。表达POU2F3的SCLC细胞系缺乏典型的神经内分泌标志物，并表现出与簇绒细胞相似的表达图谱，这表明SCLC可能存在多种起源细胞^[18]^。

### 2.4 SCLC-I

除了上述3种亚型外，还发现了一种没有表达典型转录因子，但特异性表达许多免疫检查点（immune checkpoint, IC）和人类白细胞抗原（human leukocyte antigen, HLA）基因的亚型，将其命名为SCLC-炎症或SCLC-I。

SCLC免疫治疗的临床试验^[12]^结果显示，SCLC患者极少对免疫检查点阻断（immune checkpoint blockade, ICB）药物产生反应，部分原因可能是细胞毒性T细胞等免疫细胞的低渗透。但是在SCLC-I中CD8A和CD8B的表达明显增高，说明该亚型有更多的细胞毒性T细胞浸润。虽然每个亚型都有相似的免疫细胞类型，但SCLC-I中的T细胞、自然杀伤细胞和巨噬细胞等免疫细胞群的绝对数量显著增加，且编码HLAs和其他抗原呈递机制的基因在SCLC-I亚型中的表达水平相对较高。此外，SCLC-I亚型细胞一般存在免疫检查点分子高表达的现象，包括编码程序性死亡配体1（programmed death-ligand 1, PD-L1）的CD274、编码其受体程序性细胞死亡蛋白1（programmed cell death protein 1, PD-1）的PDCD1、细胞毒性T淋巴细胞相关蛋白4（cytotoxic T lymphocyte-associated antigen-4, CTLA4）和编码CTLA4配体的CD80、CD86等；而免疫检查点CD38、IDO1、TIGIT、C10orf54（Vista）、ICOS和LAG3也经常在SCLC-I亚型中高表达；干扰素基因刺激物（stimulator of interferon genes, STING）诱导的T细胞吸引趋化因子CCL5和CXCL10在SCLC-I中也存在高表达。这些高表达的免疫分子为ICB反应提供了良好的炎症微环境。SCLC的ICB免疫治疗临床试验的失败可能与该试验未区分患者不同的SCLC亚型有关，而SCLC-I亚型患者极可能在ICB治疗中获益。

上述各SCLC亚型中，SCLC-A和SCLC-N属于NE类型，肿瘤细胞具有典型的神经内分泌特征，能够表达嗜铬粒素A（chromogranin A, ChgA）和突触素（synaptophysin, SYP）等NE标志物；而SCLC-P和SCLC-I属于Non-NE类型，不表达ChgA和SYP，但高表达REST等^[12]^。

## 3 对SCLC分子分型不同观点的探讨

Yes相关蛋白1（Yes-associated protein 1, YAP1）是Hippo生长信号通路激活的转录调节因子，在部分SCLC细胞中也存在高表达，有研究^[14]^把高表达YAP1的SCLC细胞定义为SCLC-Y亚型，但免疫组化的研究结果并不能支持将该类肿瘤作为独立亚型进行分类，测序数据也显示YAP1及其转录靶点在SCLC-P和SCLC-I两种亚型中的表达高于SCLC-A和SCLC-N亚型。

通过对目前测序数据的分析发现，几乎所有SCLC细胞都表达ASCL1、NeuroD1、POU2F3和YAP1四种转录因子中的一种或多种；或者表达SCLC-I亚型的炎症基因。原发性人SCLC组织中的甲基化组测序或RNA-seq的结果，进行一致性聚类的结果也基本都与上述四种亚型的分类结果一致。GEMM小鼠的数据^[9]^表明，MYC高表达的小鼠会向着NeuroD1-high方向发展；在条件性敲除*Trp53*/*Rb1*/*Rbl2*基因的GEMM小鼠中激活Notch通路，可以发展成ASCL1-low和NeuroD1-low的分型，并且人类肿瘤中*Notch*基因的失活突变可能会影响SCLC的亚型，导致肿瘤异质性产生。

全基因外显子组测序和100多例肿瘤全基因组测序的结果显示，SCLC中抑癌基因*TP53*和*RB1*的失活几乎普遍存在，但极少存在已知致癌基因的反复性靶向突变。研究发现MYC家族原癌基因拷贝数扩增，被抑制的表观遗传调控因子高频突变以及Notch家族失活突变等现象。这些均是SCLC治疗的靶向因子，深入研究这些靶向因子，将会在SCLC的诊断与治疗上有更大的突破。

通过对SCLC的大量研究与测序数据的分析发现，SCLC的发生与发展是由基因突变、拷贝数变异、转录组调节和表观遗传调节等多个方面共同调控的，其不同的调控因子的表达与基因的突变等均会影响SCLC的走向，形成不同的亚型。SCLC不同的分子分型更有利于前期精确诊断与治疗，有助于提高SCLC患者的存活率。但在目前人们对于SCLC的分子分型仍不太清楚，后期还需要更加深入的研究。

## 4 SCLC分子分型的临床研究

目前，SCLC的治疗包括在LS-SCLC期行手术治疗和放化疗，在ES-SCLC期行放化疗或联合ICB治疗^[19]^。随着人们对SCLC分子分型研究的逐渐深入，未来可能会提出基于此的新兴疗法。目前，DLL3抑制剂、抗体药物偶联物、双特异性T细胞诱导剂和嵌合抗原受体T（chimeric antigen receptor T, CAR-T）细胞构建物等都可用于SCLC-A的治疗。特司林-洛伐妥珠单抗（rovalpituzumab teserine, Rova-T）是一种抗体药物结合物，由一个针对DLL3的单克隆抗体、一个可拆分的蛋白酶连接物和一个吡咯环苯二氮杂卓（pyrrolobenzodiazepine, PBD）弹头组成。在针对复发性SCLC的首次人体临床试验中，尽管产生严重的副作用，但是Rova-T还是有一定的效果。然而二期和三期临床试验结果不尽人意，所以终止了Rova-T的研发^[20]^。除了DLL3抑制剂，BCL2抑制剂因其可以靶向ASCL1，也可以用于SCLC-A的治疗；但临床上SCLC患者使用BCL2抑制剂治疗的数据目前尚少^[21]^。有研究^[22]^显示，黄素腺嘌呤二核苷酸依赖性去甲基化酶LSD1（lysine-specific histone demethylase A1）在SCLC-A早期阶段存在高表达，其抑制剂可能用于SCLC-A的治疗，目前正处在早期发展阶段。

小鼠模型中的研究^[23]^发现，MYC和NeuroD1高表达的SCLC-N亚型在联合化疗时对极光激酶AURKA抑制剂具有较高的敏感性，AURKA的扩增与紫杉醇耐药有关，这也是联合紫杉醇作为该亚型二线治疗的原因之一。由于AURKA的磷酸化会导致LKB1的损伤，进而导致免疫治疗耐药；所以可以使用ICB和极光激酶抑制剂的联合发挥作用。此外，SCLC-N可能对PI3K/mTOR通路抑制剂和HSP90抑制剂也具有敏感性^[21]^。据报道^[18]^，SCLC-P细胞系对IGFR1抑制剂敏感，但该抑制剂并没有相关临床研究。

## 5 总结和展望

鉴于SCLC的恶性程度较高、进展较快，临床诊断的SCLC患者多处于ES期，存活时间较短。对SCLC进行分子分型及不同亚型的特性研究，有助于了解SCLC各不同亚型的生物学特性，确定不同亚型间的差异、共性和相互转化的能力，进而找到影响不同亚型SCLC发生、发展和转移的关键因素。将SCLC分子分型的研究应用于临床，将有助于提高医生对SCLC患者的详细诊断和治疗方案的进一步优化，从而延长患者生存时间并提高患者生活质量。
